# Compulsory admissions of patients with mental disorders: State of the art on ethical and legislative aspects in 40 European countries

**DOI:** 10.1192/j.eurpsy.2020.79

**Published:** 2020-08-24

**Authors:** D. Wasserman, G. Apter, C. Baeken, S. Bailey, J. Balazs, C. Bec, P. Bienkowski, J. Bobes, M. F. Bravo Ortiz, H. Brunn, Ö. Bôke, N. Camilleri, B. Carpiniello, J. Chihai, E. Chkonia, P. Courtet, D. Cozman, M. David, G. Dom, A. Esanu, P. Falkai, W. Flannery, K. Gasparyan, G. Gerlinger, P. Gorwood, O. Gudmundsson, C. Hanon, A. Heinz, M. J. Heitor Dos Santos, A. Hedlund, F. Ismayilov, N. Ismayilov, E. T. Isometsä, L. Izakova, A. Kleinberg, T. Kurimay, S. Klæbo Reitan, D. Lecic-Tosevski, A. Lehmets, N. Lindberg, K. A. Lundblad, G. Lynch, C. Maddock, U.F. Malt, L. Martin, I. Martynikhin, N. O. Maruta, F. Matthys, R. Mazaliauskiene, G. Mihajlovic, A. Mihaljevic Peles, V. Miklavic, P. Mohr, M. Munarriz Ferrandis, M. Musalek, N. Neznanov, G. Ostorharics-Horvath, I. Pajević, A. Popova, P. Pregelj, E. Prinsen, C. Rados, A. Roig, M. Rojnic Kuzman, J. Samochowiec, N. Sartorius, Y. Savenko, O. Skugarevsky, E. Slodecki, A. Soghoyan, D. S. Stone, R. Taylor-East, E. Terauds, C. Tsopelas, C. Tudose, S. Tyano, P. Vallon, R. J. Van der Gaag, P. Varandas, L. Vavrusova, P. Voloshyn, J. Wancata, J. Wise, Z. Zemishlany, F. Öncü, S. Vahip

**Affiliations:** 1 European Psychiatric Association, Committee on Ethical Issues, Strasbourg, France; 2 National Centre for Suicide Research and Prevention of Mental-Ill Health, Karolinska Institute, Stockholm, Sweden; 3 French Federation of Psychiatry, Paris, France; 4 Groupe Hospitalier du Havre, Université de Rouen, Rouen, France; 5 Flemish Association of Psychiatry, Kortenberg, Belgium; 6 Department of Psychiatry and Medical Psychiatry, Ghent University, Gent, Belgium; 7 University of Central Lancashire, Preston, United Kingdom; 8 Hungarian Psychiatric Association, Budapest, Hungary; 9 Department of Developmental and Clinical Child Psychology at the Institute Psychology Eotvos Lorand University, Budapest, Hungary; 10 Polish Psychiatric Association, Warsaw, Poland; 11 Department of Psychiatry, Warsaw Medical University, Warsaw, Poland; 12 Spanish Society of Psychiatry, Madrid, Spain; 13 Department of Psychiatry, School of Medicine, University of Oviedo, Oviedo, Spain; 14 Association of Psychiatrists of Spanish Association of Neuropsychiatry, Madrid, Spain; 15 Department of Psychiatry, Clinical Psychology and Mental Health, La Paz University Hospital, Universidad Autónoma de Madrid, Madrid, Spain; 16 Danish Psychiatric Association, Copenhagen, Denmark; 17 Institute of regional Health Research, University of Southern Denmark, Odense, Denmark; 18 Psychiatric Association of Turkey, Ankara, Turkey; 19 Ondokuz Mayıs Üniversitesi, Samsun, Turkey; 20 Maltese Association of Psychiatry, Attard, Malta; 21 University of Malta, Msida, Malta; 22 European Psychiatric Association Council of National Psychiatric Associations, Strasbourg, France; 23 Italian Psychiatric Association, Roma, Italy; 24 Department of Public Health, Clinical and Molecular Medicine, Università degli studi di Cagliari, Sardinia, Italy; 25 Society of Psychiatrists, Narcologists, Psychotherapists, and Clinical Psychologists from the Republic of Moldova, Chișinău, Moldova; 26 Department of State Medical and Pharmaceutical University “Nicolae Testemitanu”, Chișinău, Republic of Moldova; 27 Society of Georgian Psychiatrists, Tbilisi, Georgia; 28 Department of Psychiatry, Tbilisi State Medical University, Tbilisi, Georgia; 29 French Congress of Psychiatry, Paris, France; 30 University of Montpellier, CHRU Montpellier, Montpellier, France; 31 Department of Emergency Psychiatry and Acute Care, Lapeyronie Hospital, Montpellier, France; 32 Romanian Association of Psychiatry and Psychotherapy, Bucharest, Romania; 33 Medical Psychology Department, Iuliu Hatieganu University of Medicine and Pharmacy, Cluj-Napoca Romania; 34 Fondation Bon Sauveur, Bégard, France; 35 Belgium Professional Association of Medical Specialists in Psychiatry, Brussel, Belgium; 36 Department of Psychiatry, Antwerp University (UA), Antwerpen, Belgium; 37 Department of Psychiatry, Narcology and Medical Psychology, State University of Medicine and Pharmacy, Chișinău, Republic of Moldova; 38 German Association for Psychiatry, Psychotherapy and Psychosomatics, Berlin, Germany; 39 Clinic for Psychiatry and Psychotherapy, Ludwig-Maximilians-University Munich, Munich, Germany; 40 College of Psychiatrists of Ireland, Dublin, Ireland; 41 Department of Adult Psychiatry, Mater Misericordiae University Hospital, Dublin, Ireland; 42 Armenian Psychiatric Association, Yerevan, Armenia; 43 Medical Psychology Department, Yerevan State Mkhitar Herats Medical University, Yerevan, Armenia; 44 Institute of Psychiatry and Neuroscience of Paris (IPNP), University of Paris Paris, France; 45 Icelandic Psychiatric Association, Kopavogur, Iceland; 46 Psychiatric Department, Landspitali, University Hospital of Iceland, Reykjavík, Iceland; 47 Regional Resource Center of old age Psychiatry, AP-HP Centre – Université de Paris, Corentin-Celton Hospital, Paris, France; 48 Clinic for Psychiatry and Psychotherapy, Charité – Universitätsmedizin, Berlin, Germany; 49 Portuguese Society of Psychiatry and Mental Health, Lisbon, Portugal; 50 Institute of Environmental Health (ISAMB) of the Faculty of Medicine of the University of Lisbon (FMUL), Lisbon, Portugal; 51 Swedish Psychiatry Association, Sundsvall, Sweden; 52 North Stockholm Psychiatry, Stockholm County Medical Area (SLSO), Stockholm, Sweden; 53 Azerbaijan Psychiatric Association, Baku, Azerbaijan; 54 National Mental Health Centre, Baku, Azerbaijan; 55 Department of Psychiatry, Azerbaijan Medical University, Baku, Azerbaijan; 56 Finnish Psychiatric Association, Helsinki, Finland; 57 Department of Psychiatry, Faculty of Medicine, University of Helsinki, Helsinki, Finland; 58 Slovak Psychiatric Association, Bratislava, Slovakia; 59 Department of Psychiatry, Faculty of Medicine Comenius University and University Hospital, Bratislava, Slovakia; 60 Estonian Psychiatric Association, Tartu, Estonia; 61 Children Mental Health Centre of Tallinn Children Hospital, Tallinn, Estonia; 62 Department of Psychiatry and Psychiatric Rehabilitation, Teaching Department of Semmelweis University, Budapest, Hungary; 63 Norwegian Psychiatric Association, Oslo, Norway; 64 Department of Mental Health, Faculty of Medicine and Health Sciences, Norweigan University of Science and Technology, Trondheim, Norway; 65 Serbian Psychiatric Association, Belgrade, Serbia; 66 Psychiatric Association of Eastern Europe and the Balkans, Athens, Greece; 67 Department of Medical Sciences, Serbian Academy of Sciences and Arts, Belgrade, Serbia; 68 Psychiatric Centre of the Tallinn West Central Hospital, Tallinn, Estonia; 69 Forensic Psychiatry, Helsinki University and Helsinki University Hospital, Helsinski, Finland; 70 Adult Psychiatry, Stockholm County Medical Area (SLSO), Stockholm, Sweden; 71 Royal College of Psychiatrists, London, United Kingdom; 72 Faculty of Medicine, Psychiatry and Psychosomatic Medicine, Institute of Clinical Medicine, University of Oslo, Oslo, Norway; 73 St Loman’s Hospital, Mullingar, Ireland; 74 Russian Society of Psychiatrists, Moscow, Russian Federation; 75 First Pavlov State Medical University of St Petersburg, Saint Petersburg, Russian Federation; 76 Association of Neurologists, Psychiatrists and Narcologists of Ukraine, Kharkiv, Ukraine; 77 Institute of Neurology, Psychiatry and Narcology of the NAMS of Ukraine State Insitution, Kharkiv, Ukraine; 78 Department of Psychiatry, Universitair Ziekenhuis, Brussel, Belgium; 79 Lithuanian Psychiatric Association, Vilnius, Lithuania; 80 Lithuanian University of Health Sciences, Psychiatric Clinic, Kaunas, Lithuania; 81 Clinic for Psychiatry, University of Kragujevac, Kragujevac, Serbia; 82 Croatian Psychiatric Association, Zagreb, Croatia; 83 Zagreb School of Medicine and Zagreb University Hospital Centre, Zagreb, Croatia; 84 Slovenian Psychiatric Association, Ljubljana, Slovenia; 85 Ljubljana University Medical Centre, Ljubljana, Slovenia; 86 Czech Psychiatric Association, Prague, Czech Republic; 87 Third Faculty of Medicine, Charles University Prague, Prague, Czech Republic; 88 Institute for Social Aesthetics and Mental Health, Vienna, Austria; 89 Sigmund Freud University, Vienna, Austria; 90 St. Petersburg V.M. Bekhterev Psychoneurological Research Institute, St. Petersburg, Russian Federation; 91 Psychiatric Association of Bosnia-Herzegovina, Tuzla, Bosnia and Herzegovina; 92 Department of Psychiatry, University Clinical Center Tuzla, Tuzla, Bosnia and Herzegovina; 93 College Private Psychiatry of Bulgaria, Sofia, Bulgaria; 94 Nikola Shipkovenski Mental Health Centre, Sofia, Bulgaria; 95 Department of Psychiatry, University of Ljubljana, Ljubljana, Slovenia; 96 Netherlands Psychiatric Association, Utrecht, Netherlands; 97 Austrian Society for Psychiatry and Psychotherapy, Vienna, Austria; 98 Department of Psychiatry and Psychotherapeutic Medicine, Villach State Hospital, Villach, Austria; 99 Mental Health Centre, Horta-Guinardó, Barcelona, Spain; 100 Zagreb School of Medicine and Zagreb University Hospital Centre, Zagreb, Croatia; 101 Department of Psychiatry Pomeranian Medical University, Szczecin, Poland; 102 Association for the Improvement of Mental Health Programmes (AMH), Geneva, Switzerland; 103 Independent Psychiatric Association of Russia, Moscow, Russian Federation; 104 Belarusian Psychiatric Association, Minsk, Belarus; 105 Psychiatry and Medical Psychology Department, Belarusian State Medical University, Minsk, Belarus; 106 Center of Psychosocial Recovery, Yerevan State Medical University, Yerevan, Armenia; 107 Latvian Psychiatric Association, Riga, Latvia; 108 Department of Psychiatry and Narcology, Rīga Stradiņš University, Riga, Latvia; 109 Department of Psychiatry, Psychiatric Hospital of Athens, Athens, Greece; 110 Department of Psychiatry “Carol Davila” University of Medicine and Pharmacy, Bucharest, Romania; 111 Swiss Society of Psychiatry and Psychotherapy, Bern, Switzerland; 112 Psychosomatics and Psychotherapy Stradina Department, University of Riga, Riga, Latvia; 113 Casa de Saúde da Idanha and San José Psychiatric Clinic Instituto das Irmãs Hospitaleiras do Sagrado Coração de Jesus, Belas, Portugal; 114 Department of Neurology and Neurosurgery of Kharkiv Medical Academy of Postgraduate Education, Kharkiv, Ukraine; 115 Clinical Division of Social Psychiatry, Department of Psychiatry and Psychotherapy, Medical University of Vienna, Vienna, Austria; 116 CNWL NHS Foundation Trust, London, United Kingdom; 117 Israel Psychiatric Association, Ramat Gan, Israel; 118 Forensic Psychiatry Department, Bakirkoy Research and Training Hospital for Psychiatry, Neurology, and Neurosurgery, Istanbul, Turkey; 119 Department of Psychiatry, Ege University School of Medicine, Izmir, Turkey

**Keywords:** Awareness, communication, compulsory admission, education, ethics and human rights, European Psychiatric Association (EPA), involuntary admission, legal model, medical model, National Psychiatric Associations (NPAs), psychiatry in Europe, stigma

## Abstract

**Background.:**

Compulsory admission procedures of patients with mental disorders vary between countries in Europe. The Ethics Committee of the European Psychiatric Association (EPA) launched a survey on involuntary admission procedures of patients with mental disorders in 40 countries to gather information from all National Psychiatric Associations that are members of the EPA to develop recommendations for improving involuntary admission processes and promote voluntary care.

**Methods.:**

The survey focused on legislation of involuntary admissions and key actors involved in the admission procedure as well as most common reasons for involuntary admissions.

**Results.:**

We analyzed the survey categorical data in themes, which highlight that both medical and legal actors are involved in involuntary admission procedures.

**Conclusions.:**

We conclude that legal reasons for compulsory admission should be reworded in order to remove stigmatization of the patient, that raising awareness about involuntary admission procedures and patient rights with both patients and family advocacy groups is paramount, that communication about procedures should be widely available in lay-language for the general population, and that training sessions and guidance should be available for legal and medical practitioners. Finally, people working in the field need to be constantly aware about the ethical challenges surrounding compulsory admissions.

## Introduction

Earlier investigations show that both procedures and rates of compulsory hospital admissions of persons with mental disorders vary greatly across Europe and sometimes also within the same country [1–4]. Differences are mostly related to legal aspects and not to clinical assessments of the necessity of admission and the kind of treatment. European Psychiatric Association (EPA) guideline papers summarizing state of the art and evidence for treatment methods play a pivotal role in disseminating the state-of-the-art knowledge [5–16].

As involuntary admissions are associated with emotionally stressful circumstances and possibly with negative outcomes of the treatment [17,18], professionals, family, and patient organizations, as well as politicians, request development of European guidelines [19–21]. World Health Organization (WHO) addressed specifically the theme of involuntary admissions from a legal and technical perspectives in their guidance package on Mental Health Legislation and Human Rights [22].

Compulsory hospital admissions of patients with mental disorders are defined as when a patient is admitted to a psychiatric hospital without their consent. Compulsory admissions are a disputed but sometimes necessary medical procedure. Compulsory and involuntary admission terms have the same meaning and are both used interchangeably.

The involuntary admission procedure starts at the admission of the patient. During an involuntary admission, inherent ethical tensions between values relating to the individual patient’s autonomy, provision of adequate patient care, and community protection are intrinsically linked to enforced measures. Psychiatrists are therefore called upon to judge the necessity of involuntary intervention using the greatest available knowledge, sensitivity to the patient’s issue, and finding a solution respecting the informed consent process and patients’ decision-making capacity [23]. Therefore, while other protagonists such as family or neighbors could initiate the admission process when calling for medical practitioners or local authorities, they are not considered to be part of the admission procedure as they do not have any decision-making power.

Compulsory admissions can be analyzed through the prism of two different models, one medical and one legal. According to the medical model, involuntary admissions are considered as a health procedure, therefore, the legal authorities (judge, mayor, etc.) only have the function to control and validate the admission proposal made by medical staff. This procedure is adopted in some countries for example Finland, Greece, Italy, Norway, Spain, and Sweden.

The legal model states that the restriction of personal freedom can only be decided by judges, therefore reducing the role of health authorities. Advocates for the legal model state that the relationship between a patient and physician is strengthened when redirecting responsibilities to a legal representative [24]. Thus, legal representatives are directly involved in the decision-making process of an involuntary admission. This procedure is, for example, adopted in Germany [3].

As legal aspects are rooted in national legislations which differ between countries, contrary to the medical model which comprises more of a united view on the diagnostics and treatment in European countries [5–16, 25], it is difficult to standardize or regulate procedures on an intercountry level for compulsory admissions. One way to tackle those problems is to increase awareness by comparative studies and stimulate professional discussions between colleagues as it can lead to closing the gaps by choosing good examples when treating mentally ill patients when personal freedom is strangulated [26–28]. Stuart *et al*. [29] also brought to light the distress of carers and the need of support during and after the detention of family members and friends.

Committee on Bioethics’ Strategic Action Plan on Human Rights and Technologies in Biomedicine 2020–2025 at Directorate General of Human Rights and Rule of Law at the Council of Europe have interacted with the Ethics Committee of the EPA on promotion of voluntary care and are collecting examples of good practices in mental health care of Europe member states.

As there is little research on coercion in mental health care [30] and supporting data about how to make mental health care more consensual, the present survey was undertaken.

The aim of this survey on compulsory admissions by the Ethics Committee of the EPA in collaboration with the National Psychiatric Associations (NPAs) is to present similarities and differences concerning legal and medical procedures and reasons for compulsory admissions in 40 European countries with the purpose of contributing to further development improvements in involuntary care.

## Methods

### Definition

#### Mental disorder

Mental disorder is defined according to the American Psychiatric Association’s Diagnostic and Statistical Manual of Mental Disorders (see DSM-IV-TR; DSM-5) as “any condition characterized by cognitive and emotional disturbances, abnormal behaviors, impaired functioning, or any combination of these. Such disorders cannot be accounted for solely by environmental circumstances and may involve physiological, genetic, chemical, social, and other factors” [31].

### The survey

This report is based on a survey performed by the Ethics Committee of the EPA during 2018–2020 and approved by the EPA executive committee, the EPA board, and the council of NPAs. The survey was sent to 44 NPAs coming from 40 countries. Belgium, France, Spain, and Russia have two NPAs. Additionally, the UK has one NPA but had three respondents to the survey due to the nature of different legislation in Scotland, Northern Ireland, and Wales. Germany has slight differences in federal legislations regarding compulsory admissions.

The survey comprises 10 yes-or-no questions and 5 open-ended questions (Table 1) for 44 NPAs in 40 countries and relied on purposive sampling as every country was contacted. If clarification or more information were needed, the members of the Ethics Committee contacted the NPAs to obtain information from their respondents. All 40 countries who are member of the EPA replied to the survey.

**Table 1.** Survey questions presented in this article.

Survey	Questions	Answers	
1	Is there one or several laws regulating this process in your country? If yes, please send us the name of the law and translate the respective paragraphs.	Yes	No
2	Is there a requirement for an independent medical expert (e.g., psychiatrist not working in the respective hospital) to be involved?	Yes	No
3	Does the patient have a guaranteed right to a legal counselor to protect his/her rights?	Yes	No
4	Is a judge involved in making the final decision about compulsory admission?	Yes	No
5	Is there a time limit on legal decisions on compulsory admission?	Yes	No
6	Is a judge involved in reviewing the case after a certain period of time?	Yes	No
8	If there is a requirement for an independent medical expert (e.g., psychiatrist not working in the respective hospital), please specify what expertise is required.	Insert answer	
10 (a)	What are the usual reasons for involuntary admissions in your country? Risk to individual’s own life.	Yes	No
10 (b)	What are the usual reasons for involuntary admissions in your country? Risk to individual’s own health.	Yes	No
10 (c)	What are the usual reasons for involuntary admissions in your country? Risk for self-neglect and/or homelessness.	Yes	No
10 (d)	What are the usual reasons for involuntary admissions in your country? Risk of substantial decrease in social or occupational status due to psychotic disorder.	Yes	No
10 (e)	What are the usual reasons for involuntary admissions in your country? Risk to other people’s safety (violence…).	Yes	No
10 (f)	What are the usual reasons for involuntary admissions in your country? Risk to other people’s life.	Yes	No
11	Please list other reasons used for compulsory admission in your country.	Insert answer	

The topics that were discussed in the questionnaire can be summarized into three sections.

#### First section

Revolved around the specific laws that exist in the countries and for a description of the key participants in the compulsory admission process.

#### Second section

Concentrated on typical reasons for compulsory admission within the respondent’s respective country.

#### Third section

Not reported here due to incomplete data, was about country statistics, for example, number of psychiatric inpatients; compulsory admitted inpatients; and compulsory outpatients. Therefore, we refer the reader to the international comparative study from Sheridan Rains et al. [32] which provides data on the annual incidence of involuntary admissions for many of the countries present in the NPA survey.

### The report

Once the participants (i.e., NPAs) completed the survey, a first draft of the report was written using the quantitative categorical data gathered. A literature review on involuntary admission was performed to analyze the survey results. Keyword search in English [involuntary] [compulsory] “[admission]” “[psychiatric]” [wards] [hospital] [NPA country] on Scopus and Google Scholar databases were used. While the Scopus database was analyzed in full, constraining sampling was used for Google Scholar with 10 selected seed articles of systematic reviews of European legislation and their references until saturation was obtained [33]. In total, 347 peer-reviewed articles out of 1,093 were selected. When peer-reviewed articles were not accessible or did not exist for some countries, 12 reports and/or translated mental health legislation stemming from European governmental and nongovernmental organizations (NGOs) were included in the literature review. Once all articles were collected, a database was built and organized into two main sections, legal procedures/actors, and reasons for involuntary admissions for each individual NPA country. The analysis comparing the literature and the survey data from the 40 country respondents is presented in the results section, along with a discussion.

Preliminary results were presented during the Ethics Committee meetings in Stockholm on the September 21, 2018, and later results were discussed in Brussels on the November 25, 2019. The preliminary report was sent for feedback to the EPA Executive Committee and all NPAs in January 2020.

This report is divided into themes stemming from the survey’s questions. Each theme presents the countries answers and is followed by a discussion based on a literature review and inputs from representatives for the NPAs. Results are presented in Tables and Figures which were designed when appropriate based on the categorical data available.

## Limitations

The survey had some minor dropouts for some questions (two countries for Q10c—What are the usual reasons for involuntary admissions in your country? Risk to self-neglect and/or homelessness; Q10d—What are the usual reasons for involuntary admissions in your country? Risk of substantial decrease in social or occupational status due to psychotic disorder). Some questions were excluded in the analysis because the answers were incomplete such as Q7 (Is there any other person or professional, other than those mentioned above, involved in a compulsory admission procedure?), Q9 (If there is any other person or professional involved in a compulsory admission procedure, please specify). Moreover, some countries (like Belarus, Bulgaria, Estonia, Turkey) have less scientific literature and nongovernmental reports than other countries which was a potential limitation for discussion of the survey result. No legal expertise was involved in the survey or analyses and interpretation of the findings. All presidents of the NPAs were offered to review the manuscript.

## Results

### Legislation in Europe

All 40 countries that are members of the EPA and responded to the survey have a legislation for compulsory admissions.

### Independent medical expert in involuntary admission

The expert is defined as a medical professional who has a certain level of authority, knowledge, and expertise in the medical and or mental health field, whereas “involved” is defined as being part of the decision-making process of admitting a patient for involuntary admission. The responses to the question about requirement of medical expert in compulsory admission are presented in Table 2.

**Table 2.** Requirement for the involvement of an independent medical expert (e.g., psychiatrist not working in the respective hospital) in the compulsory admission procedure.

	Number	Countries
Yes	19	Armenia; Austria; Belgium; Bosnia and Herzegovina; Croatia; Denmark; Estonia; Finland; France; Georgia; Hungary; Ireland; Israel; Italy; Netherlands; Malta; Norway; Romania; Slovenia
No	19	Azerbaijan; Belarus; Bulgaria; Czech Republic; Greece; Iceland; Latvia; Lithuania; Moldova; Poland; Portugal; Russia; Serbia; Slovakia; Spain; Sweden; Switzerland; Ukraine; Turkey
Variation in same country	2	Germany (varies between states); United Kingdom (England, Wales, and Scotland: Yes; Northern Ireland: No)

Many countries within the European Union require more than one medical professional to be involved in the decision to involuntarily admit a patient. The matter of whom and how many assessors that should decide upon the psychiatric/medical criteria for involuntary admission is important in order to further protect the rights of patients being detained unwillingly. This measure, where more than one expert opinion is required, means that an extra precautionary step is taken in order to guarantee the rights of the patient and decrease the likelihood of abuse. Comments from some countries highlighted that even though the second medical practitioner does not have to be a psychiatrist, it is common practice to involve a psychiatrist in most cases. Italy stated that the law requires the involvement of a second medical expert but does not specify that it should be a psychiatrist who does not work in the respective hospital. The level of medical professional experience needed in the process of compulsory admission varies as is presented in Table 3.

**Table 3.** Categories of independent medical expert responsible for the compulsory admission process, answers given by 20 countries.

External medical experts	Number	Countries
Psychiatrist	16	Austria; Belgium; Bosnia and Herzegovina; Croatia; Finland; Georgia; Germany; Hungary; Iceland; Ireland; Israel; Italy; Malta; Netherlands; Romania; UK
Court expert in psychiatry	1	Slovenia
Mental health expert	1	Norway (i.e., psychologist)
Senior medical doctor	1	France
Medical doctor	5	Belgium; Denmark; Finland; Italy; Norway

### Judge in compulsory admission

Judges tend to be involved in involuntary admission procedure as Table 4 demonstrates. The records of involuntary admission and coercive measures need to always be available for a judge to review. Before authorizing the involuntary admission, judges often need to collect information from the patient, relatives, and community mental health professionals in addition to reviewing the case made by any authorities involved (psychiatrists, ward physicians, police officers, etc.).

**Table 4.** Judge involvement in making the decision about compulsory admission.

	Number	Countries
Yes	33	Armenia; Austria; Azerbaijan; Belarus; Belgium; Bosnia and Herzegovina; Bulgaria; Croatia; Czech Republic; Estonia; France; Georgia; Germany; Greece; Hungary; Iceland; Israel; Italy; Latvia; Lithuania; Moldova; Netherlands; Poland; Portugal; Romania; Russia; Serbia; Slovakia; Slovenia; Spain; Switzerland; Turkey; Ukraine
No	6	Denmark; Finland; Ireland; Malta; Norway; Sweden
Variable	1	United Kingdom (Yes: Scotland; No: England, Wales, and Northern Ireland)

In addition to having to decide on admission according to the legal model, a judge can further be included in the process to review and/or prolong the compulsory admission for a time period of 7 days to 12 months depending on the country (Table 5). Interestingly, comparing results from Tables 4 and 5 shows that Finland, Norway, and Sweden that do not have a judge involved in making the decision about involuntary admission have a judge reviewing the case, meaning that the legal system is still present for compulsory admission. Conversely, Armenia, Latvia, Poland, and Turkey which all have a judge making the decision about compulsory admission do not have a judge reviewing the case later. Whereas in Ireland, a judge is neither involved in making the final decision about compulsory admission nor involved in reviewing the case after a certain period of time. In Latvia and Slovakia, the judge is only informed of the patient discharge from the hospital. Lastly, in Italy, the judge is involved in reviewing the case, but this revision occurs only if a “renewal” of compulsory treatment is requested, which is sent by the head of the psychiatric ward to the mayor at the end of the 1-week compulsory admission duration. Even in this case, the decision of the mayor goes under the scrutiny of the judge.

**Table 5.** Involvement of a judge in reviewing the case after a certain period of time.

	Number	Countries
Yes	32	Austria; Azerbaijan; Belarus; Belgium; Bosnia and Herzegovina; Bulgaria; Croatia; Czech Republic; Estonia; Finland; France; Georgia; Germany; Greece; Hungary; Iceland; Israel; Italy; Lithuania; Moldova; the Netherlands; Norway; Portugal; Romania; Russia; Serbia; Slovakia; Slovenia; Spain; Sweden; Switzerland; Ukraine
No	7	Armenia; Denmark; Ireland; Latvia; Malta; Poland; Turkey
Variable	1	United Kingdom (Yes: England, Wales, and Scotland; No: Northern Ireland)

### Time limit on compulsory admission

Most countries have a time limit on legal decisions for compulsory admission (Table 6). Typically, this time period is short but can range from 24 h to 15 days. Norway stated that the duration of involuntary admission can be up to 1 year at a time and can even potentially extend to decades. Interestingly, in Italy, while typically the duration of compulsory admission ranges from 10–12 days, it is possible for the head of the psychiatric ward to request a renewal for an additional week. As a consequence, theoretically, compulsory admission could be renewed on a weekly basis without definite time limits if allowed by the mayor and judge.

**Table 6.** Time limit on legal decisions on compulsory admission.

	Number	Countries
Yes	38	Austria; Azerbaijan; Belarus; Belgium; Bosnia and Herzegovina; Bulgaria; Croatia; Czech Republic; Denmark; Estonia; Finland; France; Georgia; Germany; Greece; Hungary; Iceland; Ireland; Israel; Italy; Latvia; Lithuania; Malta; Moldova; the Netherlands; Norway; Poland; Portugal; Romania; Russia; Serbia; Slovakia; Slovenia; Spain; Sweden; Switzerland; Ukraine; United Kingdom
No	1	Turkey

### Right for patient to legal counselor

Table 7 presents the patient’s guaranteed right to a legal counselor, and 36 of the countries’ respondents answered positively. Patients have the right to a legal representative (lawyer) who can assist during the involuntary admission process (information and/or support often free of charge) and who can help patients appeal the decision. However, there are exceptions. In Poland and Slovakia, although a patient has the right to a legal counselor, patients (or family) would need to pay for one themselves. Additionally, family or significant others can be involved in the compulsory admission process as legal participants for the patient, which is the case in Slovakia for example.

**Table 7.** Patient’s guaranteed right to a legal counselor (lawyer).

	Number	Countries
Yes	36	Armenia; Austria; Azerbaijan; Belarus; Belgium; Bosnia and Herzegovina; Bulgaria; Croatia; Czech Republic; Denmark; Estonia; Finland; France; Georgia; Germany; Hungary; Iceland; Ireland; Israel; Italy; Latvia; Lithuania; Moldova; the Netherlands; Norway; Poland; Portugal; Romania; Russia; Serbia; Slovakia; Slovenia; Spain; Sweden; Ukraine; United Kingdom
No	4	Greece; Malta; Switzerland; Turkey

However, while the patient having legal counsel is a right, it does not mean that this right is enforced automatically. Indeed, for some countries, it is not an obligation to have the presence or consultation of a legal representative to assist the patient. The patient or family member might not be aware of this right, and medical practitioners might not be obligated to mention it. Table 8 compares the answers of the NPA present survey respondents and the participants of Sheridan Rains et al. [32] (only the countries present in both studies were included here) and highlights the differences found between guaranteed right for legal counsel and obligation for legal counsel presence during compulsory admissions (white for yes and gray for no). For example, in Finland, patients have the right to legal counsel, but this counsel is not compulsory unless patients request one. Meaning that it becomes crucial whether medical practitioners are (or are not) forced to mention this right to their patients. Table 8 shows that Austria, Belgium, and France, despite not having compulsory presence of the patient’s legal counsel, have a mandatory visit from a judge within 4–12 days.

**Table 8.** NPA survey and literature comparison about guaranteed right to legal counsel and compulsory presence for legal counsel for patient during involuntary admissions.

	Austria	Belgium	Finland	France	Germany	Italy	Norway	Portugal	Spain	Sweden	Switzerland	UK
Patient guaranteed right to legal counsel (from NPA survey)												
Patient legal counsel compulsory (presence/consultation) (from Sheridan Rains et al. [32])	No, but judge has to visit within 4 days	No, but judge has to visit within 10 days		No, but judge has to visit within 10–12 days	Not at the state level							

### Reasons for involuntary admission

**Figure 1.**
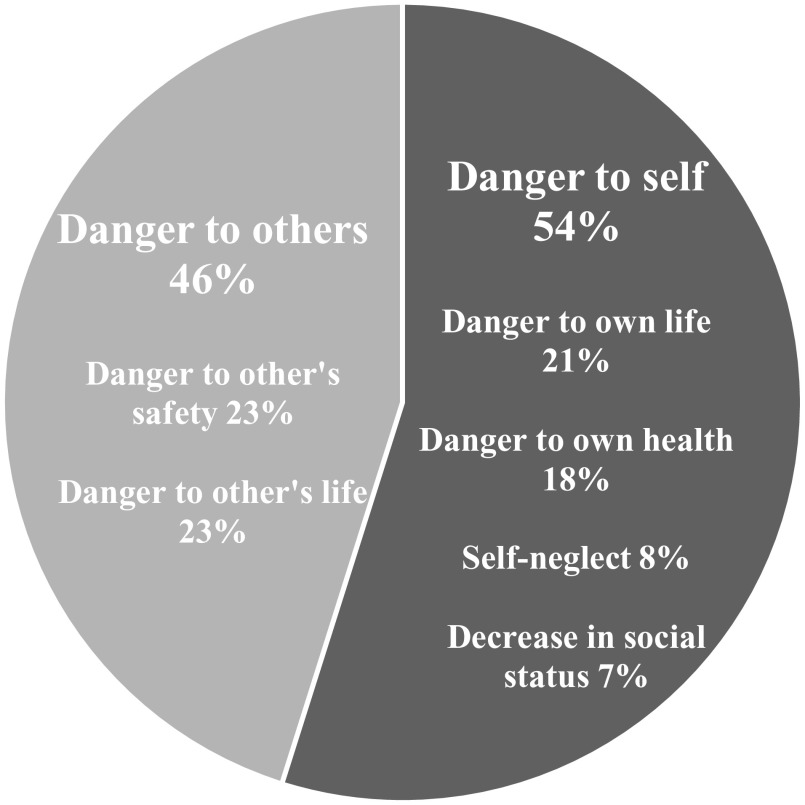
Involuntary admission reasons divided in two categories^1^.

Beside the reasons listed in Figure 1, some other reasons for which an individual could be compulsory admitted were mentioned in the survey answers. For example, inaccessibility or refusal of medication can be a reason (along with others) to be involuntary admitted in Belarus, Belgium, Finland, Ireland, Italy, and Russia. Finland and Italy can involuntary admit a patient if the outpatient procedure is not sufficient for the patient’s medical need. Third, Israel and Moldova can involuntarily admit a patient if they cause severe damages to property due to their mental state. It is interesting to note that Italy, and more recently Spain, removed the “danger to self and others” criteria from their reasons for compulsory admission.

## Discussion

### Legal aspects

Since the 1950s, mental health legislations have changed considerably throughout Europe and the world [1,34]. As public attitudes and treatment practices began to shift, many European countries reformed their legal framework for treatment and involuntary admission in order to focus on ensuring the rights and safety of mentally ill patients [1,34–36]. Across many countries, the mental health legislative schemes differ slightly, particularly regarding the criteria for admission, and who authorizes it [35,37,38]. For some countries, that is, Spain, involuntary admission regulations are included in a general law and not necessarily in a specific Mental Health Law. While most nations governing statutes, acts or regulations are applicable nationwide, Germany has slightly different legislations between states [36]. Present national mental health laws are designed with the intent of allowing governments to intervene when citizens are unable to protect their own interests, not only for the safety of individuals but also for the overall welfare of society and its members. There are several international documents which focus on Human Rights that are available as guidelines for state legislations, such as the Declaration of Hawaii from 1983 [39], the Principles for the Protection of Persons with Mental Illness [40], the Ten Basic Principles for Mental Health Law distributed by the WHO [41], and WHO Mental Health and Human Rights document [22], which are only a few examples. At the heart of all legislations is the central emphasis that the rights of patients must be protected, and involuntary treatment is only advised as an exceptional measure [3].

The literature shows that while an additional medical opinion is often involved in the process of admitting a patient, the practitioners do not have to be a “medical expert,” nor a psychiatrist, it can be an emergency doctor without specialization [1,37,38]. Further, it has been recommended by the WHO in the mental health care law: 10 basic principles that an assessment should be performed by two independent medical practitioners separately [41].

The duration of time for compulsory admission differs among nations within Europe. There is a big difference between countries in terms of the maximum amount of time that someone can be initially placed in involuntary admission. In some countries (Denmark, France, Portugal, and Spain), there is not a maximum period of time defined for compulsory admission [1,37]. Ideally, as recommended by the WHO, mental health treatments should be efficient; the amount of time in which a person is unwillingly admitted should be limited and such measure ought to be used as a last resort intervention [41].

Protecting the patient rights during an involuntary admission is paramount and is defined as a medical ethical principle. Sheridan Rains et al. [32] found that for most European countries participating in their study (Austria, Belgium, Finland, France, Germany, Italy, Norway, Portugal, Spain, Sweden, Switzerland, and the UK), patients have the right to legal counsel but that a legal representative is not required to be present or consulted during the involuntary admission. Having legal support is important as compulsory admissions can be decided upon by different civil authorities and a legal counselor can thus support the patient through the different processes [37]. This right to a legal counselor supporting the patient reported in this survey is in congruence with findings in the literature regarding the guaranteed right to appeal the involuntary admission decision [32]. During the involuntary admission process, tailored communication and information need to be given to patients. Patients need to clearly be informed about their rights, diagnosis, and treatment at every step of the process [3].

### Medical aspects

If patient autonomy and consent are key principles of medical practice, the responsibility of a state on its citizens has the power to overthrow these values, within limits [42,43]. Therefore, defining criteria for patients to be involuntary admitted is paramount to prevent abuse. Research found that the main criterion for compulsory admission to mental health care across European Union Member States is a confirmed mental disorder [1,3,37,42–45]. However, mental disorder is a necessary for most countries but not sufficient condition to be admitted. The notion of social dangerousness which Fiorillo et al. [3] defined as dangerous behavior for own life (and health) but also for others is very often highlighted as an admission criterion by the European Union Member States within different variations. This rationale can be explained this way: mental disorder causes danger (for oneself and others), the disorder can improve with a provided treatment, and thus can remove the danger [43]. But the dangerousness rationale has also been criticized as being a tool to authorize the detention of mentally ill people unlikely to become dangerous in hope to institutionalize the small number who will. These detractors of the danger criteria emphasize that focusing on the capacity of the patient to refuse/accept treatment would rehumanize involuntary admissions [46].

Italy and Spain have implemented a special legislation introducing the need-for-treatment criterion. Therefore, the psychiatrist focuses on his professional obligation to provide treatment to improve the patient’s mental health. Before, the Italian legislation’s focus was on the legal obligation to protect society from the patient [37,38,42,43]. According to several literature reviews, Sweden also added the need-for-treatment criteria into practice for involuntary admissions [37,38,42]. Finally, this is the case also for Norway, either need-for-treatment or danger (to self or others) is mandatory for involuntary admission. Still, need-for-treatment would be the condition most commonly used to prevent stigmatization [42]. Indeed, this treatment criterion leaves less room for nonmedical actors in the involuntary procedure [37].

During the analysis of the results and throughout the drafting process, several other topics came to light that are worth addressing.

### Stigma

Mental-ill health, despite being widespread in many societies, continues to be seen as taboo and a source of shame and stigma [47]. People with mental disorders are considered as vulnerable in society [48]. Patients can experience compulsory admissions as devaluating and stigmatizing. It can be stigmatizing to become an institutionalized psychiatric patient, but it can be further stigmatizing for the patient to have their normal social role taken away while being involuntarily admitted [49]. Studies have been done to assess the emotional reactions and impact on involuntary admitted patients with mental disorders. Research highlights that shame and stress due to stigma felt during involuntary admission lead to self-stigma which in turn decreases empowerment and leads to poor quality of life [50]. Xu et al. [51] demonstrated that stress related to stigma may have lasting harmful effects on recovery and called for future research on antistigma interventions to improve patient empowerment.

This survey shows that the main legal reasons for compulsory admissions are danger to other’s safety (in the survey 23%) and danger to other’s life (in the survey 23%) (see Figure 1). Therefore, the law increases the gap between involuntary admitted patients and “others” that are supposedly being put in danger and thus increases stigma. Additionally, there are many instances of involuntary admitted outpatient procedures that involve compulsory care. Patients would thus continue to be related to involuntary practices outside of the psychiatric institution, creating possibly more stigma. Therefore, we think that nonstigmatizing wording of legal procedures for compulsory admissions should be discussed as it could help decrease stigma. Changing the legislation for each country to include the need-for-treatment criterion, while still considering social determinants of mental health of the patient, would mean that a patient involuntarily admitted would be admitted for medical reasons (need-for-treatment) and not for social reasons (danger to others). While both the medical and nonmedical models exist, acknowledging and considering the benefits of either model are important. Perhaps the real choice is not between the purely medical or legal perspective but between differentially balanced models to avoid stigmatization.

### Awareness

This survey highlights that procedures for involuntary admissions and actors involved in the process varies between European countries. These variances can be exacerbated by the different cultures unique for countries and different public health approaches toward psychiatry. Additionally, there are ethnic disparities in compulsory admissions. First-generation ethnic minority groups and migrants have a higher risk of being involuntary admitted for early psychosis compared with other populations [52]. This highlights the need for culturally appropriate psychiatric practices especially during compulsory admissions and may be targeted awareness campaigns to enable medical practitioners on transcultural practices [12,24].

Additionally, more awareness is needed to improve knowledge but also society’s perceptions of involuntary admissions. Raising awareness will improve society’s knowledge about patient rights and will empower future psychiatric patients and families to take a more active role in treatment decisions and defend themselves against possible abuse. Patients in psychiatric care should be more involved in the planning, development, and evaluation of health care services, in treatment guideline formulation, research activities, and health reforms [3]. This effort needs to be multidisciplinary and involve actors from all of the decision-making chain as well as patients and family advocacy groups, like Global Alliance of Mental Illness Advocacy Networks (GAMIAN) and European Federation of Associations of Families of People with Mental Illness (EUFAMI).

### Communication

Communication is central to care. The results of this survey are in line with the findings of the EUNOMIA study concerning the role of information and communication to protect patients’ rights during involuntary hospitalization [3]. Healthcare providers and legal procedure actors need to be able to communicate with psychiatric service users and their families [3,11,28]. The terms and processes related to compulsory admission in both legislation and medical documents should be communicated in an understandable way for patients to know their rights and reasons for admission. In the case of compulsory admission especially, the rights of the patient are of the utmost importance. This includes basic human rights but also right to legal counsel. Therefore, if the legislation, or any formal medical documents related to the process, cannot be clearly comprehended by the lay person, then this could allow room for misunderstanding and negligence. It is also very important for the legislation to be easily accessible and understandable for the patient, families, and involved medical and/or legal professional staff. Some organizations provide such information in a way that is both easily available and not difficult to understand, for example, the charity Mind in England and Wales which provides advice and support regarding involuntary admission to people experiencing any mental health–related issues. With that being said, the responsibility of ensuring that people know their rights should not fall solely on such organizations. It would be beneficial to include patient and family advocacy groups and organizations, as well as governmental and NPAs, in the process of facilitating the understanding of the legal language currently used in legislations and other formal documents. It is especially important to communicate clearly with the patients and the family as well as having available information on compulsory admission considering that the family can be a legal participant and advocate for the patient. This information should then be available online and in leaflets located in relevant institutions for patients, families, and the general public.

## Conclusions

The report highlights the role of nonmedical actors in the involuntary admission procedures such as legal counsels, judges, or prosecutors as well as the movement toward the use of the need-for-treatment criteria. Creating seminars, courses, and writing guidelines for legal and medical practitioners, prepared with input from patients and family organizations, will stimulate use of good practices in compulsory admissions and promotion of voluntary treatment. All training should account for the role of gender, cultural, social, and religious/spiritual factors in the mental state of patients

## Data Availability

EPA Strasbourg Office, France.

## Footnotes

1 Detailed answers of the NPAs are available but are not included in this article
